# A comparison of pediatric and adult CT organ dose estimation methods

**DOI:** 10.1186/s12880-017-0199-3

**Published:** 2017-04-26

**Authors:** Yiming Gao, Brian Quinn, Usman Mahmood, Daniel Long, Yusuf Erdi, Jean St. Germain, Neeta Pandit-Taskar, X. George Xu, Wesley E. Bolch, Lawrence T. Dauer

**Affiliations:** 1 Department of Medical Physics, Memorial Sloan Kettering Cancer Center, 1275 York Avenue, Box 84, New York, NY 10065 USA; 2 Department of Radiology, Memorial Sloan Kettering Cancer Center, 1275 York Avenue, New York, NY 10065 USA; 3 Department of Mechanical, Aerospace, and Nuclear Engineering, Rensselaer Polytechnic Institute, Troy, NY 12180 USA; 4 J. Crayton Pruitt Family Department of Biomedical Engineering, University of Florida, Gainesville, FL 32611 USA

**Keywords:** CT dosimetry, Organ dose, Effective dose, Pediatric, Adult, Monte Carlo

## Abstract

**Background:**

Computed Tomography (CT) contributes up to 50% of the medical exposure to the United States population. Children are considered to be at higher risk of developing radiation-induced tumors due to the young age of exposure and increased tissue radiosensitivity. Organ dose estimation is essential for pediatric and adult patient cancer risk assessment. The objective of this study is to validate the VirtualDose software in comparison to currently available software and methods for pediatric and adult CT organ dose estimation.

**Methods:**

Five age groups of pediatric patients and adult patients were simulated by three organ dose estimators. Head, chest, abdomen-pelvis, and chest-abdomen-pelvis CT scans were simulated, and doses to organs both inside and outside the scan range were compared. For adults, VirtualDose was compared against ImPACT and CT-Expo. For pediatric patients, VirtualDose was compared to CT-Expo and compared to size-based methods from literature. Pediatric to adult effective dose ratios were also calculated with VirtualDose, and were compared with the ranges of effective dose ratios provided in ImPACT.

**Results:**

In-field organs see less than 60% difference in dose between dose estimators. For organs outside scan range or distributed organs, a five times’ difference can occur. VirtualDose agrees with the size-based methods within 20% difference for the organs investigated. Between VirtualDose and ImPACT, the pediatric to adult ratios for effective dose are compared, and less than 21% difference is observed for chest scan while more than 40% difference is observed for head-neck scan and abdomen-pelvis scan. For pediatric patients, 2 cm scan range change can lead to a five times dose difference in partially scanned organs.

**Conclusions:**

VirtualDose is validated against CT-Expo and ImPACT with relatively small discrepancies in dose for organs inside scan range, while large discrepancies in dose are observed for organs outside scan range. Patient-specific organ dose estimation is possible using the size-based methods, and VirtualDose agrees with size-based method for the organs investigated. Careful range selection for CT protocols is necessary for organ dose optimization for pediatric and adult patients.

## Background

Besides the natural background, medical exposure is the largest source of ionizing radiation exposure to the human population [1, 2]. Computed Tomography (CT) is one of the most widely adopted medical imaging modalities in clinical use, and is increasingly used because of the technology advancements and the improvements in medical infrastructure [1, 3–5]. CT scans contribute up to 50% of the medical exposure to the United States (US) populations in 2006 [1, 2]. The annual number of CT examinations in the US has increased by 10% each year from 1993 through 2011, up to 85 million in 2011, and stabilized around 80 million with 0.6 million annual change at most since 2011 [6]. With a population of 325 million in the US in 2016 where 24% of the population is pediatrics and adolescents under age of 18, one in four Americans has a CT scan each year [7]. The high number of CT scans and high contribution of CT scans to medical exposure raised concerns in the radiation protection and radiology community. The International Commission on Radiological Protection (ICRP) addressed the importance of multi-detector CT patient dose management in 2007 [4]. The principles of optimization and ‘as low as reasonably achievable’ (ALARA) have been major principles and have been adopted in the radiation protection of patients, the public, and radiological workers for decades [8–11]. The American College of Radiology (ACR) introduced the Dose Index Registry in 2011 to facilitate the collection and comparison of the CT dose indices for all participating medical entities [12]. A group of radiologists formed the Alliance for Radiation Safety in Pediatric Imaging & the Image Gently Alliance, and started the Image Gently campaign in 2007 to send a message of reducing the amount of radiation when performing pediatric CT scans [13]. For adults, ACR introduced the Image Wisely campaign in 2010 to raise awareness of eliminating unnecessary exams as well as using only the amount of radiation necessary for required image quality [14].

Children are generally considered to be at higher risks of developing radiation-induced tumors because of the young age of exposure and increased tissue radiosensitivity in some of the organs [15–17]. For the 23 types of cancers reviewed recently by the United Nations Scientific Committee on the Effects of Atomic Radiation (UNSCEAR) committee, children are clearly more likely to develop one of a quarter of these types, including leukemia, brain, breast, skin, and thyroid cancer [15]. However, for the other three quarters of the cancer types, children are no more sensitive (such as colon cancer or lung cancer) than adults, or there is either not enough data or no clear relationship between radiation exposure and cancer risks [15]. The UNSCEAR committee recommends avoiding the use of generalized radiation risks for children and emphasizes the evaluating of and using specific organ dose, the importance of which has been recognized by the radiology community with respect to radiation induced cancer risk estimations [15, 18–24].

Organ dose is the absorbed dose to a specific organ in the body, and is generally estimated as the ratio of the amount of ionizing radiation energy deposited in the organ to the mass of the organ, representing an estimate of the average damage to the organ per unit mass. Organ dose is not a dose estimate that is readily available to radiologists or physicians in clinical CT scans. Rather, the CT scanners commonly report volumetric CT dose index (CTDI_vol_) and dose length product (DLP) at the end of examinations [25]. CTDI_vol_ represents the average radiation dose for a standardized CTDI phantom over the entire field of view and through a scan length of 100 mm along the longitudinal axis after taking the pitch of the scan into consideration [25]. DLP is the integrated dose for the entire CT scan length, and is equal to the product of CTDI_vol_ and scan length [25]. It is clear that either CTDI_vol_ or DLP, when CTDI_vol_ is an average radiation dose estimate and DLP is an overall radiation dose estimate to standardized phantoms, is not a good estimate of patient organ dose. Size-specific dose estimates (SSDE) were introduced in 2011 to adjust the CTDI_vol_ to address the effect of the patient sizes on the average radiation dose, especially for small-size pediatric patients and large-size overweight patients [26]. Methods and recommendations of the calculations and usage of SSDE were updated in a later publication [27], but the quantity itself remained a poor estimate for individual organ dose that did not account for the tissue differences or the geometric location of the organ [22, 23, 28].

Although organ dose cannot be directly measured on living tissues or organs, measurements in physical anthropomorphic phantoms are possible. However, they require great amounts of time, equipment, and skilled staff to perform [19, 29–35]. A practical method of accurate organ dose estimation is to use Monte Carlo (MC) methods and anatomically realistic computational anthropomorphic phantoms to simulate the CT scans and to calculate the organ doses [18, 20, 21, 23, 36, 37]. Sophisticated computation codes such as MCNPX incorporate the Monte Carlo method and can be used to model the CT scanner and simulate the transport of ionizing radiation in anthropomorphic phantoms [38–40]. Unlike stylized phantoms which are composed of three dimensional geometric objects such as spheres and cylinders, computational anthropomorphic phantoms resemble the realistic anatomical features of patient morphologies and faithfully apply the compositions of the body tissues according to standards or reference sets [41–43]. Thus, the use of realistic phantoms generates more accurate dose results than using stylized phantoms [20, 42, 44–46]. Pediatric patient phantoms, pregnant patient phantoms, and adult patient phantoms with various body sizes were developed to address the age, pregnancy, or body size variations among patient populations [47–51].

Various MC-based organ dose calculators can be currently acquired, allowing quick dose calculations for medical physicists and physicians. Most of the widely used calculators are based on the unrealistic stylized phantoms, such as ImPACT and CT-Expo [52, 53]. CT-Expo integrated two adult phantoms and two pediatric phantoms, allowing for some representative pediatric organ dose estimations [53]. However, ImPACT provides no intrinsic calculation method for pediatric organ dose estimation, while supplying a set of ranges of adjustment factors for roughly estimating effective dose to pediatric patients [52]. A few newly developed dose calculators utilize anatomically realistic phantoms and provide better patient-matching options for organ dose calculation. VirtualDose is the first online organ dose and effective dose calculator that incorporates anatomically realistic phantoms for patients of various ages (including pediatric ages 0 through 15), gender, pregnancy stages, or body sizes [23].

The objective of this study is to validate the VirtualDose software in comparison to currently available software and methods for pediatric and adult CT organ dose estimation. First, CT-Expo and VirtualDose are used to generate the major portion of the organ dose data. Then, ImPACT is used to calculate and compare adult organ doses as it lacks specific pediatric phantoms [52]. Thirdly, body-size based MC methods for organ doses of patients of various sizes are also investigated, and compared with the doses by VirtualDose [18, 22, 24, 28]. Finally, pediatric-to-adult effective dose ratios are also calculated with VirtualDose and compared to the ranges of the effective dose ratios provided by ImPACT. Additionally, the effect of the scan range change on organ dose is discussed to show the importance of scan range selection on dose optimization.

## Methods

Organ dose and effective dose for pediatric patients who received CT scans were calculated with three dose calculators: VirtualDose, CT-Expo, and ImPACT. Four CT protocols were investigated: head, chest, abdomen-pelvis (AP), and chest-abdomen-pelvis (CAP). With VirtualDose, pediatric patients at 5 different age groups were covered: 0-year-old, 1-year-old, 5-year-old, 10-year-old, and 15-year-old. Adult patients of normal sizes were also included in the calculations. Organ doses by VirtualDose were also compared to the organ doses based on size-dependent functions from literature [18, 22, 24, 28]. The ratios of the effective doses of the 5 pediatric groups to the normal size adults were calculated and compared to the ranges of pediatric-to-adult effective dose ratios by ImPACT.

### CT protocols

Four CT protocols were simulated in the study to cover the head and the trunk of patients: head, chest, AP, and CAP. Since the dose calculator VirtualDose provided the largest collection of pediatric phantoms, the scan range defined in VirtualDose for the four protocols was also applied to CT-Expo and ImPACT as best as possible. For head protocol, the scan range was from the top of head through C1 lamina. For chest protocol, the scan range was from the clavicles through the diaphragm. For AP protocol, the scan range was from the top of liver through the pubic symphysis. For CAP protocol, the scan range was from the clavicles through the pubic symphysis. No over-scan was taken into account, as a pitch of 1 was used. A Siemens Somatom Sensation 16 CT model, which was the scanner model employed in the Monte Carlo simulations of the pediatric phantoms [23], was used in the calculation of dose data for the three dose calculators. In VirtualDose, for 0-year-old, 1-year-old, 5-year-old, and 10-year-old patients, head bowtie filters were used in all four protocols. For 15-year-old and adult patients, head bowtie filters were used for head protocols, and body bowtie filters were used for other protocols. The rest of CT scan parameters were kept the same for all protocols and all phantoms to enable more direct comparisons: 120 kVp tube voltage, 100 mAs tube current time product, a pitch of 1, and 10 mm beam collimation. The effective dose was calculated using tissue weighting factors from ICRP No. 103 publication employing the gender-average methodology [10].

### Organ dose calculators

VirtualDose was a web-based CT organ dose and effective dose calculator that incorporated 25 “virtual patient” phantoms covering pediatric patients, pregnant patients, normal size adult patients, and overweight adult patients [23]. The 5 pairs of male and female pediatric phantoms covering 0-year-old, 1-year-old, 5-year-old, 10-year-old and 15-year-old patients were used in this study, in addition to a pair of normal size male and female adults. The doses to 15 organs to which tissue weighting factors were assigned in the ICRP No. 103 Publication, as well as doses to the 13 organs defined as remainder in the report and the effective dose, were estimated [10]. The CTDI_vol_ was 16.6 mGy for the protocols using head bowtie filters, and it was 6.8 mGy for the protocols using body bowtie filters. Organ dose and effective dose were normalized with these CTDI_vol_ values accordingly, to reduce the scanner dependency [54]. The scan range of the four protocols simulated by VirtualDose was listed in Table 1. For 0-year-old, 1-year-old, 5-year-old, and 10-year-old patients, the scan range were the same between males and females.

**Table 1:** Scan range for VirtualDose in this study

Patient	Height	Head		Chest		AP^a^		CAP^b^	
		Start	End	Start	End	Start	End	Start	End
0YM	47.5	39.8	47.1	28.0	35.8	13.5	29.6	13.5	35.8
0YF	47.5	39.8	47.1	28.0	35.8	13.5	29.6	13.5	35.8
1YM	76.5	64.4	75.6	47.8	58.4	28.7	49.0	28.7	58.4
1YF	76.5	64.4	75.6	47.8	58.4	28.7	49.0	28.7	58.4
5YM	110.5	96.0	108.8	75.9	89.4	50.0	76.9	50.0	89.4
5YF	110.5	96.0	108.8	75.9	89.4	50.0	76.9	50.0	89.4
10YM	140.5	125.2	138.6	100.0	117.8	66.7	102.0	66.7	117.8
10YF	140.5	125.2	138.6	100.0	117.8	66.7	102.0	66.7	117.8
15YM	166.5	152.0	165.0	117.4	140.4	81.8	120.6	81.8	140.4
15YF	161.7	147.8	159.8	114.8	136.2	80.2	118.1	80.2	136.2
RPIM	176.0	164.0	176.0	124.0	151.0	86.8	124.8	86.8	151.0
RPIF	163.5	152.5	163.0	115.0	140.0	82.0	115.1	82.0	140.0

^a^ *AP* abdomen-pelvis, ^b^CAP, chest-abdomen-pelvis

Note: both scan range and phantom height are in unit of cm. The simulated CT scans start from inferior location (Start) through superior location (End). The bottom of the feet of phantoms is defined as 0 cm

CT-Expo was a Microsoft Excel based application for patient CT dose calculation, and used the dose evaluation methods mentioned in CT exposure surveys in Germany [53, 55]. The application was capable of reporting organ doses and effective doses using the tissue weighting factors of the ICRP No. 103 Publication [10]. However, the application only included 4 stylized patient phantoms: one for adult male (ADAM), one for adult female (EVA), one for children at age of seven (CHILD), and one for infants (BABY) [53, 56]. The doses for 31 organs and tissues were available, but for comparison purposes the 28 organ doses available in VirtualDose were also collected in CT-Expo. The average of the lower large intestine dose and the upper large intestine dose was considered as the colon dose for calculations with CT-Expo. Due to lacking anatomical details in organs and tissues of the stylized phantoms, the scan range was matched on these phantoms as best as possible, and the start and end locations were listed in Table 2. The location of pubic symphysis was surrogated by the location of the bottom of the trunk, and the location of C1 vertebrae was approximated by the location where the cylindrical spine intercepts the oval head.

**Table 2:** Scan range for CT-Expo in this study

Patient	Head		Chest		AP		CAP	
	Start	End	Start	End	Start	End	Start	End
BABY	29	38	14	25	0	18	0	25
CHILD	47	63	28	42	0	32	0	42
ADAM	81	94	41	71	-2	43	-2	71
EVA	75	89	38	67	0	40	0	67

Note: both scan range and phantom height are in unit of cm. The simulated CT scans start from inferior location (Start) through superior location (End). The trunk base of the phantoms is defined as 0 cm

Several comparisons of the estimated organ dose by VirtualDose to the estimated organ dose by CT-Expo were made. The 0-year-old and 1-year-old doses of VirtualDose were compared to the BABY doses of CT-Expo. The 5-year-old and 10-year-old doses of VirtualDose were compared to the CHILD doses of CT-Expo. The 15-year-old and adult doses of VirtualDose were compared to the adult doses of CT-Expo. The comparisons were performed for both males and females, although for patients younger than 10-year-old there were no differences in doses to most organs between males and females except for doses to gonads. Organs that were outside the scan range and with doses smaller than 0.5 mGy were not included in dose comparisons as the inherent errors of the doses might be comparable to the doses themselves. Effective doses calculated with tissue weighting factors from ICRP No.103 publication were also included in the comparisons, and were noted as ED103 in figures [10]. Two-sample *t*-test was performed for a list of in-field organs in each scanned region between VirtualDose and CT-Expo for 0-year-old (or BABY), 5-year-old (or CHILD), and adult. For head scan, the organ list included the brain and the lens of eye. For chest scan, the organ list included the breast, the esophagus, the lungs, and the thymus. For AP scan, the organ list includes the colon, the liver, the stomach, the urinary bladder, the adrenals, the gall bladder, the kidneys, the pancreas, the small intestine, the spleen, and the uterus (female)/prostate (male). For CAP scan, the organ list was the combination of the lists of chest scan and AP scan. For each of the aforementioned scan region, the null hypothesis was the VirtualDose doses and the CT-Expo doses were from distributions of equal means and equal variances. In each *t*-test, the dose lists of 0-year-old, 5-year-old, and adult for both male and female were concatenated into one list for VirtualDose, and the dose lists of BABY, CHILD, and adult were concatenated into one list for CT-Expo before the test was performed on the resultant lists of the two software. Similar *t*-test was also performed for effective dose of the two by concatenating the effective dose results across patients and scan regions into one list for each tool and using the resultant lists in the test. Statistical significance was defined as *p* < 0.05.

ImPACT was also a Microsoft Excel based spreadsheet application for patient CT dose calculation, and used the Monte Carlo data by the National Radiological Protection Board (NRPB) in the United Kingdom [52]. The application only included the MIRD hermaphrodite adult phantom, and employed adjustment factors for the effective dose of pediatric patients [57, 58]. For adults, organ doses were calculated using the ImPACT spreadsheet and the scan range for ImPACT calculations was listed in Table 3. The adult organ doses were compared between VirtualDose, CT-Expo, and ImPACT. Ratios of VirtualDose to CT-Expo, and ratios of VirtualDose to ImPACT were calculated and demonstrated for the simulated head CT scan and the simulated CAP CT scan. Effective doses with tissue weighting factors from ICRP No.103 publication were also compared between the three codes [10]. Two-sample *t*-test was performed between VirtualDose and ImPACT, VirtualDose and CT-Expo, and ImPACT and CT-Expo for adult. The included in-field organs were the same as the ones defined previously for head scan and CAP scan. The organ doses of the two scan regions and both genders were concatenated into one list for each tool before the test was performed on the resultant lists. The null hypothesis was the doses of the two compared tools were from distributions of equal means and equal variances. Statistical significance was defined as *p* < 0.05.

**Table 3:** Scan range for ImPACT in this study

Patient	Head		CAP	
	Start	End	Start	End
MIRD	80	94	-1	71

Note: both scan range and phantom height are in unit of cm. The simulated CT scans start from inferior location (Start) through superior location (End). The trunk base of the phantoms is defined as 0 cm

In addition, the ratios of pediatrics effective dose to adult effective dose were provided in the form of ranges in ImPACT spread sheet, allowing rough estimation of pediatric effective dose from adult effective dose for head and neck (HN), chest, and AP CT scans [52]. Similar pediatric-to-adult effective dose ratios were calculated with VirtualDose using the tissue weighting factors of the ICRP No. 103 Publication [10]. The ratios calculated by VirtualDose were compared to the ranges of ratios by ImPACT.

### Body-size based methods

The organ doses of patients were affected by the size of the body region being scanned. Besides the SSDE metric introduced by AAPM, several groups investigated the effects of the body sizes on organ doses, and developed empirical functions [18, 21, 22, 24, 28]. Turner et al. found a strong exponential relationship between body-CTDI_vol_-normalized organ doses and patient perimeter of the abdominal region [18]. Care must be taken in utilizing the exponential function proposed by Turner et al., because typically one would apply head bowtie filter for pediatric CT exams and calculate head-CTDI_vol_-normalized organ doses as in the following studies by three other groups [18]. Tian et al. found the head-CTDI_vol_-normalized organ doses decreased exponentially with the patient diameter increasing [21, 28]. Kost et al. calculated the diameters of patients by assuming a cylindrical volume of the scanned region and performed exponential regression for head-CTDI_vol_-normalized organ doses as a function of these diameters [22]. Papadakis et al. developed exponential equations for head-CTDI_vol_-normalized organ doses as a function of the water equivalent diameters of the scanned regions of patients [24]. In this study, we applied size parameters of the pediatric phantoms in VirtualDose to the methods proposed by the aforementioned four groups and calculated the doses for a limited number of organs. The reasons for the limited number of organs are: Turner et al. only reported doses to several organs of abdominal region [18]; only the effective diameters of abdominal regions of the pediatric phantoms in VirtualDose were available; the effective diameters could be assumed to be the same as the water equivalent diameters for abdominal region [27]. We calculated the absolute absorbed doses to six organs (adrenals, kidneys, liver, pancreas, spleen and stomach) using the methods by the four groups with either effective diameters or the derived perimeters for abdomen-pelvis scans of pediatric patients [18, 22, 24, 28]. To obtain absolute absorbed doses, the head CTDI_vol_ of the Siemens Sensation 16 scanner was applied to the normalized organ doses calculated with three of the four methods, and the body CTDI_vol_ was applied to the normalized organ doses calculated with the method by Turner et al [18]. Organ doses calculated with VirtualDose were compared to the doses calculated with the four methods. Two-sample *t*-test was performed for dose to each organ across patients of various ages with a null hypothesis that VirtualDose results and results of a size-based method were from distributions of equal means and equal variances. Statistical significance was defined as *p* < 0.05. The tests were carried out for four times for each comparison of VirtualDose and one of the four size-based methods.

### Effective dose estimation for pediatrics based on adult doses

Effective dose was the sum of the gender-averaged weighted organ dose equivalents using recommended tissue weighting factors from ICRP for the purpose of estimating the dose and risk to the population being irradiated [8–10]. In this study we used the tissue weighting factors from the ICRP No. 103 publication [10]. Khursheed et al. calculated the ratios of the effective dose for pediatrics to the effective dose of adults by Monte Carlo simulations of a family of six stylized phantoms representing pediatrics and adults [58]. The range of the ratios was adopted by ImPACT for users to estimate pediatric effective doses [52]. However, ranges of ratios were difficult to use in practice. In addition, these ratios were derived based on unrealistic stylized phantoms that lack anatomical details in geometrically simplified organs, while the phantoms in VirtualDose were created based on patient CT images [23, 47, 59]. Considering such ratios as quick adjustment factors of effective doses for clinic applications, these ratios were calculated in this study with VirtualDose using the anthropomorphic pediatric phantoms and were compared with the ratio range in ImPACT [52].

## Results

### Comparison of VirtualDose and CT-Expo

**Fig. 1:**
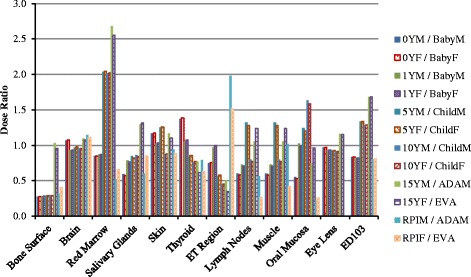
Comparisons of organ doses^*^ and effective doses between VirtualDose and CT-Expo: Head CT scan with 120 kVp tube voltage; ET region stands for extrathoracic region (nose, mouth, pharynx, larynx), and ED103 stands for effective dose with ICRP 103 tissue weighting factors [10]. *Note: Organs outside scan range and with dose smaller than 0.5 mGy are not included in Figs. 1, 2, 3, 4, 5 and 6, since the statistical error in the Monte Carlo results for these organs are high and can be as high as the dose itself

For head CT scan, the doses to eleven organs as well as the effective dose are compared between VirtualDose and CT-Expo, as demonstrated in Fig. 1. For distributed organs such as bone surface, red marrow, skin, lymph nodes, and muscle, VirtualDose results are within 0.3 and 2.7 times of CT-Expo results, where consistently significant differences are found for bone surface dose and red marrow dose among various patients. The skin doses are within 30% difference between the two codes. The lymph nodes dose is approximated with the muscle dose in CT-Expo, so it shares similar trend with the muscle dose. For organs inside the scan range such as brain and eye lens, VirtualDose results are within 0.9 and 1.2 times of CT-Expo results. However, the *t*-test shows the VirtualDose results are different from the CT-Expo results with a *p*-value of 0.0011 (*p* < 0.05). For organs partially in the scan range or outside the scan range such as salivary glands, thyroid, ET (extrathoracic) region, and oral mucosa, VirtualDose results are within 0.2 and 2.0 times of CT-Expo results. The effective doses calculated by VirtualDose are within 0.8 and 1.7 times of the effective doses calculated by CT-Expo across various patients.

**Fig. 2:**
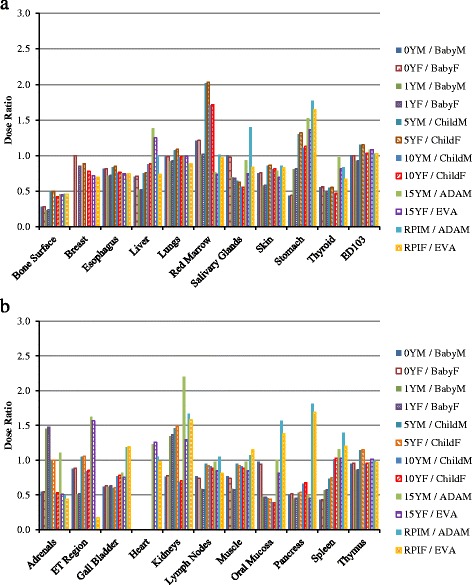
Comparisons of organ doses and effective doses between VirtualDose and CT-Expo: Chest CT scan with 120 kVp tube voltage; the results are broken into subfigure **a**) and **b**) for ease of display; ET region stands for extrathoracic region (nose, mouth, pharynx, larynx), and ED103 stands for effective dose with ICRP 103 tissue weighting factors [10]

For chest CT scan, the doses to twenty-one organs as well as the effective doses are compared between VirtualDose and CT-Expo, as demonstrated in Fig. 2. For distributed organs such as bone surface, red marrow, skin, lymph nodes, and muscle, VirtualDose results are within 0.2 and 2.0 times of CT-Expo results. Again, large differences are found in bone surface doses and red marrow doses among various patients. For organs within scan range such as breasts, esophagus, lungs, heart, and thymus, VirtualDose results are within 0.7 and 1.3 times of CT-Expo results. The *t*-test of this scan region shows that there are no statistically significant differences between VirtualDose and CT-Expo in-field organ doses with a *p*-value of 0.26 (*p* > 0.05). One should note that CT-Expo does not provide breast doses for males and in this study the male breast dose is assumed to be the same as female breast dose, and it does not provide heart doses for pediatrics either. Thus, only the female breast doses and the adult heart doses are obtained from CT-Expo and used for comparison. For organs partially in the scan range or outside the range such as liver, salivary glands, stomach, thyroid, adrenals, ET region, gall bladder, kidneys, oral mucosa, pancreas, and spleen, VirtualDose results are within 0.2 and 1.8 times of CT-Expo results. For effective doses, VirtualDose results are within 0.9 and 1.2 times of CT-Expo results.

**Fig. 3:**
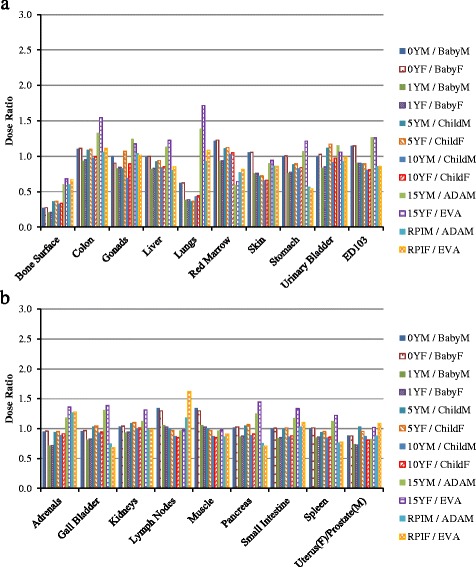
Comparisons of organ doses and effective doses between VirtualDose and CT-Expo: Abdomen-Pelvis (AP) CT scan with 120 kVp tube voltage; the results are broken into subfigure **a**) and **b**) for ease of display; ED103 stands for effective dose with ICRP 103 tissue weighting factors [10]

For abdomen-pelvis scan, the doses to eighteen organs as well as the effective doses are compared between VirtualDose and CT-Expo, as demonstrated in Fig. 3. For distributed organs such as bone surface, red marrow, skin, lymph nodes, and muscle, VirtualDose results are within 0.2 and 1.6 times of CT-Expo results, where large discrepancies occur for bone surface doses across various patients. For organs within the scan range such as colon, liver, stomach, urinary bladder, adrenals, gall bladder, kidneys, pancreas, small intestine, spleen, and uterus (female)/prostate (male), VirtualDose results are within 0.7 and 1.6 times of CT-Expo results. The *t*-test of this scan region shows that there are no statistically significant differences between VirtualDose and CT-Expo in-field organ doses with a *p*-value of 0.92 (*p* > 0.05). For the organs partially in the scan range or outside the scan range such as gonads and lungs, VirtualDose results are within 0.4 and 1.7 times of CT-Expo results. For effective doses, VirtualDose results are within 0.8 and 1.3 times of CT-Expo results.

**Fig. 4:**
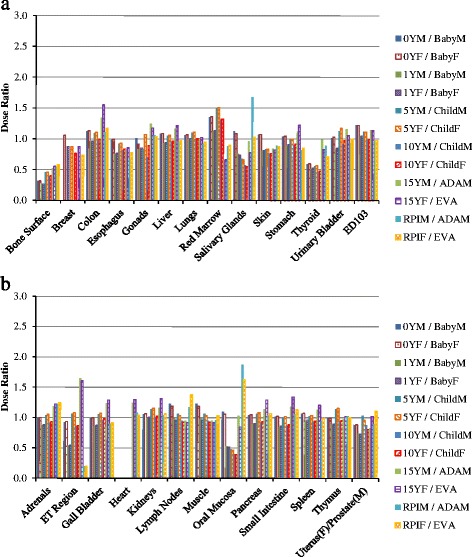
Comparisons of organ doses and effective doses between VirtualDose and CT-Expo: Chest-Abdomen-Pelvis (CAP) CT scan with 120 kVp tube voltage; the results are broken into subfigure **a**) and **b**) for ease of display; ET region stands for extrathoracic region (nose, mouth, pharynx, larynx), and ED103 stands for effective dose with ICRP 103 tissue weighting factors [10]

For chest-abdomen-pelvis scan, the doses to twenty-six organs as well as the effective doses are compared between VirtualDose and CT-Expo, as demonstrated in Fig. 4. For distributed organs such as bone surface, red marrow, skin, lymph nodes, and muscle, VirtualDose results are within 0.3 and 1.5 times of CT-Expo results, where large discrepancies exist for bone surface dose and red marrow dose across patients of various ages. For organs partially in the scan range or outside the range such as male gonads, salivary glands, thyroid, ET region, and oral mucosa, VirtualDose results are within 0.2 and 1.9 times of CT-Expo results. The rest of the twenty-six organs are within the scan range, where VirtualDose results are within 0.7 and 1.6 times of CT-Expo results. The *t*-test of this scan region shows that there are no statistically significant differences between VirtualDose and CT-Expo in-field organ doses with a *p*-value of 0.30 (*p* > 0.05). For effective doses, VirtualDose results are within 0.98 and 1.22 times of CT-Expo results. The *t*-test of the effective doses from all scans shows that there is no statistically significant difference between VirtualDose and CT-Expo with a *p*-value of 0.83 (*p* > 0.05).

### Comparison of VirtualDose, CT-Expo, and ImPACT for adults

**Fig. 5:**
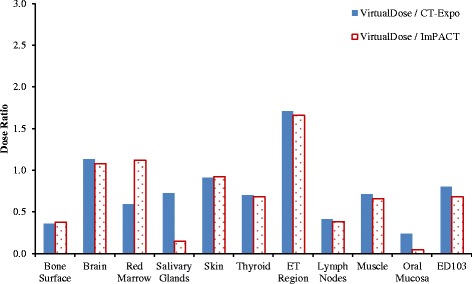
Comparisons of adult gender-averaged organ doses and effective doses among VirtualDose, CT-Expo, and ImPACT: Head CT scan with 120 kVp tube voltage; ET region stands for extrathoracic region (nose, mouth, pharynx, larynx), and ED103 stands for effective dose with ICRP 103 tissue weighting factors [10]

For head scan, the doses to ten organs as well as effective dose are compared. In general the dose ratios of VirtualDose to ImPACT are similar to the ratios of VirtualDose to CT-Expo, except for red marrow, salivary glands and oral mucosa, as shown in Fig. 5. For distributed organs and between VirtualDose and ImPACT, VirtualDose results are within 0.4 and 1.7 times of ImPACT results. For brain in the scan range, VirtualDose results are within 1.08 and 1.13 times of ImPACT results. For organs partially in the range or outside the range, VirtualDose results are within 0.2 and 1.7 times of ImPACT results. For the effective doses, the difference between VirtualDose result and ImPACT result is 32%.

**Fig. 6:**
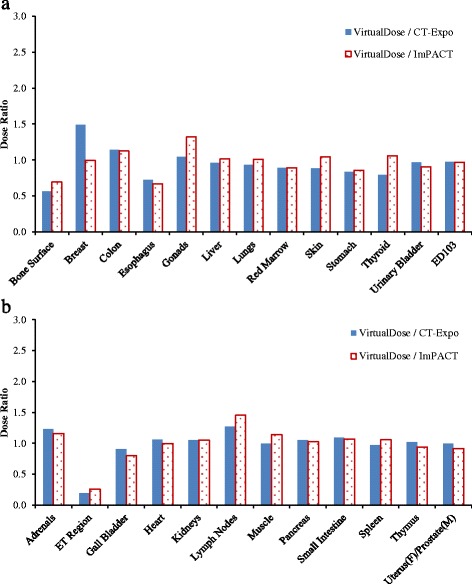
Comparisons of adult gender-averaged organ doses and effective doses among VirtualDose, CT-Expo, and ImPACT: CAP CT scan with 120 kVp tube voltage; the results are broken into subfigure **a**) and **b**) for ease of display; ET region stands for extrathoracic region (nose, mouth, pharynx, larynx), and ED103 stands for effective dose with ICRP 103 tissue weighting factors [10]

For CAP scan, the doses to twenty-four organs as well as the effective doses are compared as shown in Fig. 6. For distributed organs and between VirtualDose and ImPACT, VirtualDose results are within 0.7 and 1.5 times of ImPACT results. For organs within the scan range, VirtualDose results are within 0.7 and 1.2 times of ImPACT results. For organs partially in the range or outside the range, VirtualDose results are within 0.3 and 1.3 times of ImPACT results. For effective dose, the difference between VirtualDose result and ImPACT result is 3%.

The breast dose ratio of VirtualDose to ImPACT is different from the ratio of VirtualDose to CT-Expo, where the VirtualDose breast dose is very close to the ImPACT breast dose while the VirtualDose breast dose is 49% higher than the CT-Expo breast dose. Besides breast dose, the ImPACT doses of several organs (gonads, skin, thyroid, and lymph nodes) are different from the CT-Expo doses by more than 15%, even though both codes use stylized phantoms for dose calculations. Between VirtualDose and ImPACT, the *t*-test shows there are no statistically significant differences with a *p*-value of 0.96 (*p* > 0.05). Between VirtualDose and CT-Expo, the *t*-test shows the tools are statistically different for adult head scan and CAP scan with a *p*-value of 0.0054 (*p* < 0.05). Between CT-Expo and ImPACT, the *t*-test also shows the tools are statistically different with a *p*-value of 0.0009 (*p* < 0.05).

### Comparison of VirtualDose and body-size based methods

**Fig. 7:**
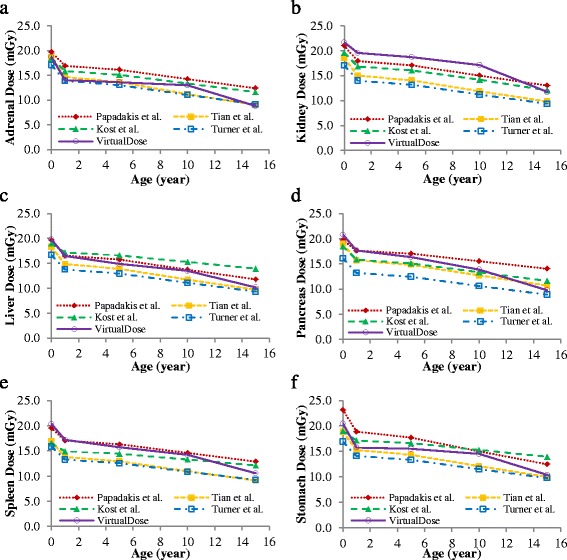
Comparison of VirtualDose with the size-based empirical functions: Organ doses for **a**) Adrenals, **b**) Kidneys, **c**) Liver, **d**) Pancreas, **e**) Spleen, and **f**) Stomach in AP CT scans [18, 22, 24, 28]

The organ doses by the four different empirical functions are compared to the VirtualDose results for abdomen-pelvis scan of pediatric patients at ages of 0-year-old, 1-year-old, 5-year-old, 10-year-old, and 15-year-old, as shown in Fig. 7. As patient age decreases, the organ doses show consistent increasing trends for all methods. The organ doses for 0-year-old patients are 1.4 to 2.1 times of the doses for 15-year-old patients, given the same scan parameters. Across the five methods, the variations of organ doses are smaller than 16% for adrenals, 17% for liver, 18% for pancreas, 16% for spleen, and 16% for stomach. Across the five patient ages, the largest variations are observed for 15-year-old patients (18%), and the smallest variations are for 0-year-old patients (5%). The doses by VirtualDose are within the dose range of the four size-based methods, except for kidneys where VirtualDose results are higher than other methods for patients under 10 years old. The doses estimated with Tian et al. method and Turner et al. method are generally relatively low for the six organs studied and the five age groups of patients, while the doses with Papadakis et al. method, with Kost et al. method, and with VirtualDose are generally relatively high [18, 21, 22, 24, 28]. Table 4 shows that VirtualDose was not statistically different (*p* > 0.05) for all the six organs from any of the four size-based methods.

**Table 4:** Two-sample *t*-test *p*-values from comparisons of VirtualDose (VD) to size-based methods for six organs of pediatric patients underwent simulated abdomen-pelvis CT scan

Compared methods	Adrenal	Kidney	Liver	Pancreas	Spleen	Stomach
VD and Papadakis et al. [24]	0.30	0.67	0.82	0.63	0.82	0.41
VD and Tian et al. [28]	0.93	0.12	0.56	0.64	0.21	0.60
VD and Kost et al. [22]	0.55	0.36	0.46	0.71	0.47	0.58
VD and Turner et al. [18]	0.71	0.054	0.31	0.16	0.14	0.30

### Effective dose adjustment factors for pediatrics based on adult doses

The ratios of the pediatric effective doses to the adult effective doses are calculated for the five age groups of phantoms in VirtualDose and for three types of scans: HN, chest, and AP, as shown in Table 5. HN scans are assumed to start at the top of skull through the level of clavicles. The ratios increase as the patients become younger, and range from 1.0 to 1.5 for HN scan, from 1.1 to 2.0 for chest scan, and from 1.5 to 2.9 for AP scan. For HN scan, the ratio changes by no more than 10% until patient is younger than 1 year old.

**Table 5:** Relative effective doses for pediatric patients

Patients	Head & neck	Chest	AP
Adult	1.0	1.0	1.0
15 y	1.0	1.1	1.5
10 y	1.1	1.5	2.0
5 y	1.3	1.6	2.2
1 y	1.4	1.8	2.3
Newborn (0 y)	1.5	2.0	2.9

Note: The relative effective doses are calculated against the effective doses for adults

**Fig. 8:**
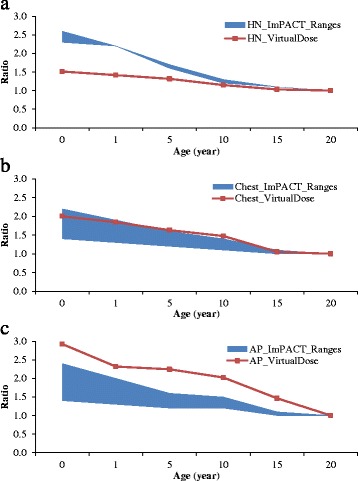
Ratios of pediatric effective doses to adult effective doses: VirtualDose ratios compared to ratio ranges provided in ImPACT for **a**) Head and Neck (HN) scans, **b**) Chest scans, and **c**) AP scans, where adults are assumed to be 20 years old, and ratio equals to pediatric effective dose (mSv) divided by adult effective dose (mSv)

Compared to the ranges of pediatric to adult effective dose ratios provided in the ImPACT spreadsheet, the factors by VirtualDose ratios are lower than the range for HN scans, within the range for chest scans, and above the range for AP scans, as shown in Fig. 8. For 0-year-old patients and HN scans, the VirtualDose ratio is below 0.65 times of effective dose ratios derived from the ranges provided in the ImPACT spreadsheet. For 5-year-old patients and AP scans, the VirtualDose ratio is above 1.38 times of effective dose ratios derived from the range of ImPACT sheet.

## Discussion

Fast and accurate estimation of organ doses for patients, especially for pediatric patients, are essential for radiologists and radiation protection professionals in clinical practice. In this study we compared existing methods of CT dose calculations for pediatric and adult patients by various groups.

Two sets of software that enable fast organ dose estimations in a few clicks of the computer mouse are compared in the beginning: VirtualDose and CT-Expo. VirtualDose is inherently more preferable to CT-Expo in that it includes more pediatric phantoms that can represent wider patient ages, and that it utilizes anatomically realistic phantoms for dose calculations. In addition, CT-Expo does not provide male breast dose at all, or heart dose to pediatric patients [53]. Four CT protocols are simulated to cover most of the radiosensitive organs in patient body. The comparisons of the results by the two pieces of software show large discrepancies as expected; with the results between each software deviating up to five times from each other. Across the four protocols, the bone surface doses by VirtualDose are consistently smaller than the doses for CT-Expo by about 70%. The mathematical phantoms used in CT-Expo do not have specific representations of the bone surface, so calculations based on such phantoms approximate the bone surface dose with the dose to the entire skeleton [60]. Such approximation explains the over-estimated bone surface doses by CT-Expo. The mathematical phantoms do not possess explicit red bone marrow models and approximate the red marrow dose by applying correction factors to dose to the whole bones, while the anthropomorphic phantoms explicitly model the spongiosa of bones for red bone marrow dose calculations [47, 59, 60]. Due to the anatomical differences between anthropomorphic phantoms and mathematical phantoms, large differences exist for red marrow dose between VirtualDose and CT-Expo, where VirtualDose results can be two times higher than the CT-Expo results.

Dose estimates to organs inside the scan range vary less between various methods than dose estimates to organs at the edge of the scan range or outside the scan range. Even between two generations of phantoms, the mathematical phantoms and the anthropomorphic phantoms, at various patient ages our study show dose differences within 60% for organs inside scan range. Between VirtualDose and CT-Expo, differences up to 5 times can occur for organs outside scan range, such as the ET region dose in CAP scans. The doses to these outside organs are contributed by scattered photons, and are typically one or two magnitudes smaller than doses to organs inside the scan range [18, 61]. In addition, large statistical errors exist in doses to outside organs from Monte Carlo simulations without high enough number of photons simulated [61].

**Fig. 9:**
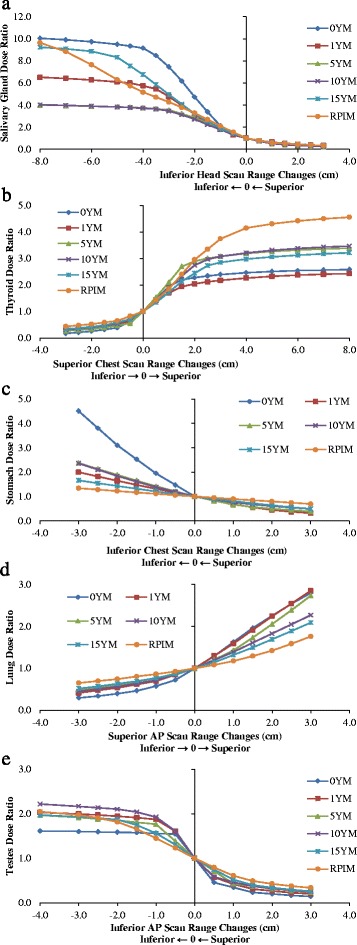
The sensitivity of doses of partially scanned organs in respect to scan range change: Head scans: **a**) Salivary Gland; Chest scans: **b**) Thyroid and **c**) Stomach; AP scans: **d**) Lungs and **e**) Testes; the arrows in horizontal axis title indicate the scan range change in the corresponding direction leads to increase in dose

Doses to the organs at the edge of the scan range are subject to the definitions of scan range, and are sensitive to changes of scan range by centimeters or even by millimeters. Additional calculations were performed using VirtualDose to determine the magnitudes of dose sensitivity of organs at the edges of scan range. The inferior edges of head protocols, the superior and inferior edges of chest protocols, and the superior and inferior edges of AP protocols are moved by 0.5 cm steps for 3cm superiorly and then 3cm inferiorly. The dose sensitivity to changes in scan range are investigated for five representative organs in male patients: the salivary gland for head scans, the thyroid at superior edges of chest scans, the stomach at inferior edges of chest scans, the lungs at superior edges of AP scans, and the testes at inferior edges of AP scans, as shown in Fig. 9. In addition, for relatively small organs such as the salivary gland, the thyroid, and the testes, the scan range is further extended such that the inflection points (beyond which the organs are less sensitive to scan range changes) of the curves are shown in the figure. Further range extension beyond inflection points impact less on the doses of the partially scanned organs, where plateau of slowly increasing dose ratios are observed.

Salivary glands are located in the jaws of the lower part of the head, and are at the edges of the head (Brain) CT scans simulated in this study. Extension of the scan range inferiorly includes more or even the entire glands into the head scans. The dose to the glands can be 7.5 times of the default dose after an inferior extension of 3 cm for new-born (0-year-old) patients. For older patients, a 3-cm extension can still increase the dose to the glands by more than 2 times. With a 1-cm inferior extension or a 1-cm superior retraction, the dose to the glands can be doubled or halved, indicating the salivary gland dose is very sensitive to the location of the inferior edge of the head scan. Comparing the doses with 3cm superior retraction and the doses with 3cm inferior extension of the inferior range, the changes in salivary gland dose can be 27 times for newborn patients and 13 times for adults. The inferior extension of head scan range increases the salivary dose, and the extension is up to 8cm to show the inflection points on the dose ratio curves. For patients under 10 years old, a 4cm inferior range extension is enough to show the inflection points. For 15-year old patients and adult patients, because of the relative large size of the gland, the organ remains sensitive to scan range change until relative large extension is made (a 5cm extension for 15-year-old, and an 8cm extension for adults).

The thyroid gland lies at the levels of the fifth cervical vertebrae through the first thoracic vertebrae of patient body, and can be partially covered in the chest CT scan. In our calculations the chest scans ended at the level of clavicles, which position at levels of the first and the second vertebrae of the body. As expected, the 3cm superior extension of the chest scan range increases the thyroid dose by up to 2.7 times for adult patients. For pediatric patients less than 5 years old the thyroid is completely covered after a 2-cm increase in scan range superiorly, and the increase in thyroid dose is small for any further range extensions. The thyroid dose is sensitive to the location of the superior edge of the chest scan, as the dose can be doubled or halved for a 1-cm change in the location. Comparing the doses with 3cm superior extension and the doses with 3cm inferior retraction of the superior range, the changes in thyroid dose can be 14 times for newborn patients and 8.5 times for adults. The superior extension of chest scan range increases the thyroid dose, and the extension is up to 8cm to show the inflection points as well as the plateau on the dose ratio curves. For all patients, a 4cm inferior range extension is enough to show the inflection points. For patients under 10 years old, the thyroid is less sensitive to scan range change after a 2cm inferior range extension.

The stomach sits inferiorly to esophagus, diaphragm and lungs, and it can be partially included in the chest CT scans at the inferior ends of the scan range. The stomach dose increases as the chest scan range are extended inferiorly, and for new-born patients the stomach doses can be 3.5 times higher with a 3 cm inferior range extension. For adult patients, however, the changes in scan range by 3cm do not have great impact on the stomach dose, where the dose changes are smaller than 34%. With 1cm change in range, the stomach dose can change by 90% for newborn patients but only 21% for adult patients. Comparing the doses with 3cm superior retraction and the doses with 3cm inferior extension of the inferior range, the changes in stomach dose can be 14 times for newborn patients but only 2 times for adults.

For AP scans, the lung doses and the testes doses are analyzed regarding to changes in scan range. The lungs are large organs in the chest cavity and can be partially included in the superior ends of the AP scans. The lungs receive more scattered photons than small organs such as salivary glands, and the lung doses are not as sensitive as salivary gland doses in regarding to the scan range changes. A 3cm increase in superior ends of range lead to 180% increase in dose for new-born patients and 76% increase in dose for adult patients. A 1cm change in scan range can lead to 60% change in dose for new-born patients, but the change can only lead to less than 17% change in dose for adult patients. Comparing the doses with 3cm superior extension and the doses with 3cm inferior retraction of the superior range, the changes in lung dose can be 9.5 times for new-born patients and 2.5 times for adults.

The testes are male gonads inferior to the pubic symphysis, and they can be potentially partially covered in the inferior end of the AP scans. In our default simulations we included part of the testes inside the scan range. As a result, the scan range changes seem to have relatively low effects on the testes doses, where a 3cm inferior extension of the scan range only increases the dose by 120% across patients of various ages. However, one should note that by comparing the doses with 3cm inferior extension and the doses with 3cm superior retraction, the changes in the testes doses can be 11 times for new-born patients and 6 times for adult patients. The inferior extension of AP scan increases the testes dose, and the extension is up to 4cm to show the inflection points on the dose ratio curves. For patients under 10 years old, the testes are less sensitive to scan range change after just a 1cm inferior range extension.

Overall the doses to organs partially included in the scan range are subject to specific scan range in practices, where 1cm change in range can lead to 60% change in dose to large organs such as stomach and lungs for newborn patients and 100% change in dose to small organs such as thyroid and salivary glands. The organs in adults are in most times less sensitive to scan range changes, and the organs in other pediatric patients are more sensitive than adults but less sensitive than newborn patients. One should note that for small organs such as thyroid and testes, dose changes of 5 times or more can occur in only a 2 cm scan range extension or retraction, especially for pediatric patients. For adults the effect of scan range changes on organ doses may not fully manifest itself until more than 3 cm changes have occurred. For example, the 3cm superior extensions of chest scan range lead to greater changes for the thyroid doses of adults than these of pediatrics. In addition, one should note that the dose ratios are calculated against the default scan range, where the five discussed organs are already partially covered in the scan range. If the organs are not included in the scan range and scan range extension is made to begin to cover such organs, more drastic dose increases should be expected. Moreover, the high sensitivity of doses of small organs to scan range implies high impact of overscan on doses to these organs when volumetric helical CT scans are performed with large beam collimations.

The comparisons between VirtualDose and CT-Expo, and the comparisons between VirtualDose and ImPACT illustrate that anatomical differences between anthropomorphic phantoms and mathematical/stylized phantoms as well as calculation methodology differences lead to large discrepancies between organ doses calculated by these tools. Across patients of various ages the statistical tests show that VirtualDose does not differ from CT-Expo significantly except for head scans. However, when comparing results for adults the *t*-test shows that VirtualDose is different from CT-Expo. Between VirtualDose and ImPACT, the *t*-test shows that the two are not different for adult head and CAP CT scan in-field organ dose estimation. Even between CT-Expo and ImPACT doses to several organs such as salivary glands and breasts are different as the scan range cannot be exactly the same due to the modifications made to the stylized phantoms [62, 63]. The *t*-test shows that CT-Expo is statistically different from ImPACT for adult head and CAP CT scan in-field organ dose estimation. The methods based on realistic anthropomorphic phantoms should be considered more preferable, as the software with stylized phantoms either do not provide direct pediatric dose calculations, or are lack of a variety of pediatric phantoms that can represent newborn, child, and adolescent pediatric patients [52, 53]. Besides the unrealistic geometries of stylized phantoms, crude approximations are made for bone surface and red bone marrow dose calculations, and doses to a few organs such as male breasts and pediatric heart are not available [52, 53].

VirtualDose provides 5 age groups of pediatric phantoms for organ doses calculations for pediatric patients. Four different research groups proposed size-based organ dose functions that were based on Monte Carlo calculations of simulated CT scans on several anthropomorphic phantoms or even tens of phantoms. The comparisons of the doses to several organs inside abdominal regions show that VirtualDose is within relatively small variations (less than 20%) of the four comparison methods. If the size parameters such as perimeters, effective diameters, or water equivalent diameters are available for specific patients, it is reasonable to use the size-based methods to estimate patient specific organ doses. However, one has to decide among the different methods, which do not match each other exactly and have variations of about 20%. More importantly, the size parameters are normally not readily available and currently require trained staff to measure them on patient CT images. Statistical analysis shows that VirtualDose is not different from the size-based methods for the organs investigated. In the cases when such parameters are not available and fast organ dose calculations are required to response to patients’ questions, VirtualDose can be the tool that conveys the dose estimates in seconds.

The effective dose ratios of pediatrics to adults by VirtualDose share similar trends with the range of ratios provided by ImPACT, although the magnitudes of the ratios are different between the two codes. For HN scans, the VirtualDose ratios are lower than the ImPACT ratios, especially for small pediatric patients. For chest scans, the VirtualDose ratios are within 10% of the ratios of ImPACT. For AP scans, the VirtualDose ratios are higher than the ImPACT ratios. The anatomical differences between the anthropomorphic phantoms used in VirtualDose and the stylized phantoms used in ImPACT have likely caused the differences. In addition, the ImPACT effective doses are normalized by air kerma before the ratios of effective doses are calculated, while no such normalizations are performed when calculating the effective dose ratios with VirtualDose.

A limitation of this study was that no physical measurement was involved and it was not practical to determine if one method was more accurate than another. In addition, the calculations in this study were performed on only a few virtual patients and it was hard to obtain enough data for statistical testings. Measurements on physical human phantoms are planned to validate the computational methods based on experiment design in literature [34, 35, 64–66]. Future work involves the application of the methods discussed in this study to a number of adult and pediatric patients for organ doses and effective dose estimation.

## Conclusion

VirtualDose has been validated in comparison to two different organ dose estimation tools and four size-based methods for pediatric and adult patients. Up to five times discrepancies in doses to organs outside the scan range or distributed organs are found between VirtualDose and the other two tools (CT-Expo and ImPACT). For organs inside scan range, the differences are smaller than 60% and may not be statistically significant. The size-based methods require patient size information such as patient diameters, and can provide estimations of organ doses for specific patients. The organ doses generated using VirtualDose are within 20% of such size-based methods and show no significant difference.

ImPACT spread sheet and CT-Expo can provide organ dose estimation for average-size adult patients, and CT-Expo can provide organ dose estimations for 7-year-old and new born pediatric patients. VirtualDose can provide organ dose estimation for pediatric patients from new-born to 15 years old and for adults. Patient-specific organ dose can be estimated with the size-based methods and the patient-specific size information, but one has to acquire the size information. Finally, one should be careful about the calculations of doses to organs partially involved in the scan range, as even change in scan range of just 2 cm can lead to a 5 times difference in doses to such organs for pediatric patients. Careful range selection for CT protocols is necessary for organ dose optimization for pediatric and adult patients.

## Abbreviations

0YF: 0 year old female
0YM: 0 year old male
10YF: 10 years old female
10YM: 10 years old male
15YF: 15 years old female
15YM: 15 years old male
1YF: 1 year old female
1YM: 1 year old male
5YF: 5 years old female
5YM: 5 years old male
AAPM: American Association of Physicists in Medicine
ACR: American College of Radiology
ALARA: As low as reasonably achievable
AP: Abdomen and pelvis
CAP: Chest, abdomen and pelvis
CT: Computed tomography
CTDI: Computed tomography dose index
DIR: Dose Index Registry
DLP: Dose length product
ED103: Effective Dose calculated with tissue weighting factors from ICRP Publication 103
ET: Extrathoracic
HN: Head and neck
ICRP: International Commission on Radiological Protection
MC: Monte Carlo
NCRP: National Council on Radiation Protection and Measurements
NRPB: National Radiological Protection Board
OECD: Organisation for economic co-operation and development
RPIF: Rensselaer Polytechnic Institute Adult Female
RPIM: Rensselaer Polytechnic Institute Adult Male
SSDE: Size-specific dose estimates
UNSCEAR: United Nations Scientific Committee on the Effects of Atomic Radiation
US: United States
VD: VirtualDose
